# A systematic review on the state-of-the-art and research gaps regarding inorganic and carbon-based multicomponent and high-aspect ratio nanomaterials

**DOI:** 10.1016/j.csbj.2024.10.020

**Published:** 2024-10-19

**Authors:** Anastasios G. Papadiamantis, Angelos Mavrogiorgis, Stavros Papatzelos, Dimitris Mintis, Georgia Melagraki, Iseult Lynch, Antreas Afantitis

**Affiliations:** a NovaMechanics Ltd., Nicosia, Cyprus; b School of Geography, Earth, and Environmental Sciences, University of Birmingham, UK; c Entelos Institute, Larnaca, Cyprus; d Division of Physical Sciences and Applications, Hellenic Military Academy, Vari, Greece

**Keywords:** Systematic review, Research gaps, Inorganic multicomponent, Carbon-based multicomponent, High-aspect ratio, Nanomaterials

## Abstract

This review explores the state-of-the-art with respect to multicomponent nanomaterials (MCNMs) and high aspect ratio nanomaterials (HARNs), with a focus on their physicochemical characterisation, applications, and hazard, fate, and risk assessment. Utilising the PRISMA approach, this study investigates specific MCNMs including cerium-zirconium mixtures (Ce_x_Zr_y_O_2_) and ZnO nanomaterials doped with transition metals and rare earth elements, as well as Titanium Carbide (TiC) nanomaterials contained in Ti-6Al-4V alloy powders. HARNs of interest include graphene, carbon-derived nanotubes (CNTs), and metallic nanowires, specifically Ag-based nanowires. The review reveals a significant shift in research and innovation (R&I) efforts towards these advanced nanomaterials due to their unique properties and functionalities that promise enhanced performance across various applications including photocatalysis, antibacterial and biomedical uses, and advanced manufacturing. Despite the commercial potential of MCNMs and HARNs, the review identifies critical gaps in our understanding of their environmental fate and transformations upon exposure to new environments, and their potential adverse effects on organisms and the environment. The findings underscore the necessity for further research focused on the environmental transformations and toxicological profiles of these nanomaterials to inform Safe and Sustainable by Design (SSbD) strategies. This review contributes to the body of knowledge by cataloguing current research, identifying research gaps, and highlighting future directions for the development of MCNMs and HARNs, facilitating their safe and effective integration into industry.

## Graphical abstract

**Figure:**
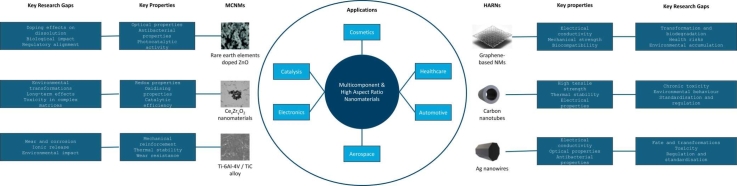

## 1 Introduction

Micro/nanoelectronics and photonics, life sciences, advanced materials, advanced manufacturing, artificial intelligence (AI), and security and connectivity are the 6 key enabling technologies on which Europe is focussing its research and innovation (R&I) [1]. These technologies, coupled with rapid computational advancements, drive innovation across different industries providing positive socioeconomic impact and growth for Europe’s citizens. Based on the latest study published by the European Union Observatory for Nanomaterials (EUON) [2], nanomaterials (NMs) will play a key role in all of the abovementioned sectors, with technological advances and public demand for functional, lightweight, and affordable state-of-the-art products driving growth [2]. The European (EU, EEA, and Switzerland) NM market is expected to grow at a compound annual growth rate (CAGR) of 13.9 % per volume and 18.4 % per value, reaching 271.29 Kilotons and 12.6 € billions by 2025, respectively [2].

**Fig. 1:**
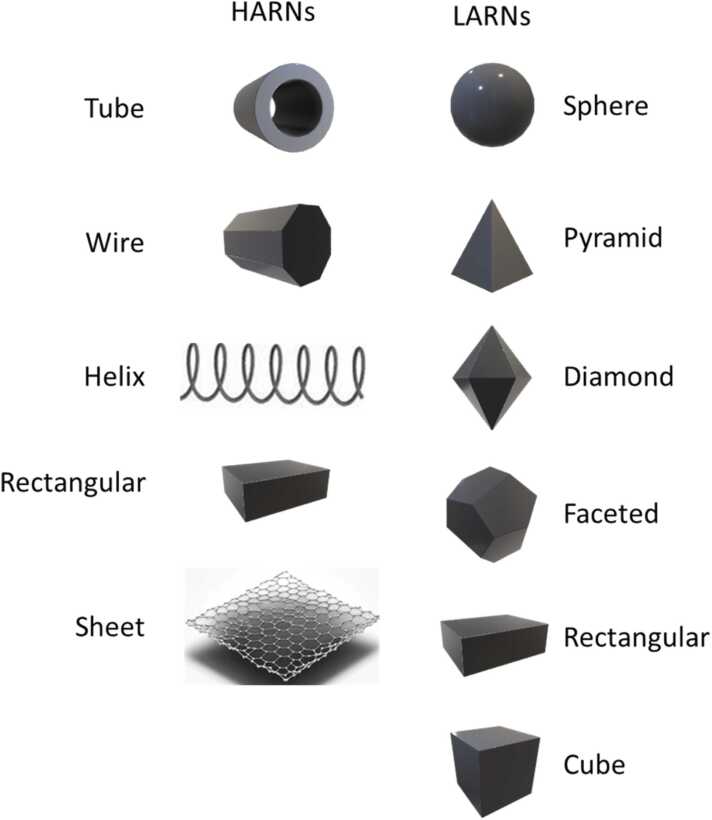
HARNs vs. traditional low aspect ratio NMs (LARNs).

While traditional NMs, e.g., metals and metal oxides, are currently the leading market segment [2], the need for more functional NMs with unique properties has shifted R&I efforts towards more advanced and complex NMs. Among those, multicomponent NMs (MCNMs), as defined in [3], and high aspect ratio NMs (HARNs), as defined in [4], have started to play a key role. MCNMs are complex multicomponent, and hybrid NMs demonstrating novel properties and functionalities [5]. HARNs (see Fig. 1 for comparison of certain HARNs with traditional low aspect ratio NMs) refer to NMs such as nanotubes, nanowires, and nanosheets including 2D NMs like graphene, which “*have two similar external dimensions and a significantly larger third dimension (aspect ratio of 3:1 or greater) and substantially parallel sides*” [4].

Considering the usefulness and potential benefits of MCNMs and HARNs, several publicly funded projects support the adoption of MCNMs and HARNs from industry. These projects are working towards understanding the potential effects of MCNMs and HARNs based on their exposure, release, fate, and hazard characteristics. In this way, the development of Safe and Sustainable by Design (SSbD) strategies that industry, and especially Small and Medium Enterprises (SMEs), will be able to adopt and implement in their manufacturing process, are promoted. To achieve operationalisation of SSbD for advanced materials, deep understanding of the state-of-the-art research on the hazard and fate assessment of MCNMs and HARNs following release and exposure is required to enable risk assessment. Similarly, appreciation of the limitations of the methods used to assess these materials is necessary to ensure proper interpretation of the data. For traditional NMs these topics have already been extensively reviewed [6], [7], [8], and are beyond the scope of this paper. The challenge regarding MCNMs and HARNs is that most studies, so far, have been focussed on pristine or simple NMs, while potential transformations of the NMs when exposed to new environments are still not being routinely monitored as part of their physicochemical characterisation even though it is well understood that it is these transformed forms that typically interact with living organisms [9], [10], [11]. Similarly, the potential adverse effects of MCNMs and HARNs to organisms and the environment will originate following their release and/or exposure and will, potentially, be accompanied by respective transformations and changes in their properties and behaviour. The lack of insights into the impacts of transformed MCNMs and HARNs hinders their wider commercialisation in nano-containing products, as regulators lean towards a more precautionary approach regarding granting approval for such materials and nano-containing products.

The purpose of this systematic review, catalogued using the PRISMA approach [12], is to identify the state-of-the-art regarding research on selected representative MCNMs and HARNs with key applications in the cosmetics, health, automotive, aerospace, oil and gas, and electronics sectors. For MCNMs these include cerium-zirconium mixtures (Ce_x_Zr_y_O_2_), which have applications, among other, as catalytic converters into the automotive industry [13], [14], ZnO loaded with transition metals and rare earth elements for use in cosmetics, e.g., sunscreens [15], and health, e.g., bioimaging, drug delivery [16], and Titanium Carbide (TiC) NMs contained in Ti-6Al-4V alloy powders for use in the automotive and aerospace industries as lightweight alternatives to conventional materials [17], [18]. For HARNs the selected materials include graphene and its derivatives used as composites in the automotive [19], oil and gas [20], and textile industries [21], carbon nanotubes (CNTs) used in high-performance structures/components [22], and metallic nanowires, i.e., Ag-based nanowires for electronic applications, e.g., printed electronics [23]. While it is expected that these materials have been studied in their pristine forms, studies regarding their properties and behaviour when integrated into complex matrices are lacking. Thus, this review is focuses on research regarding the properties and behaviour of these materials in complex environments rather than on specific applications.

## 2 Methodology

### 2.1 Protocol and registration

The current review was performed using the Systematic Reviews and Meta-Analyses (PRISMA) checklist [12]. The review question was: “*What is the state-of-the-art regarding the safety and/or toxicity of complex multicomponent and/or high aspect ratio nanomaterials, as represented by cerium-zirconium mixtures (Ce*_*x*_*Zr*_*y*_*O*_*2*_*), or ZnO loaded with transition metals or rare earth elements, or Titanium Carbide (TiC) nanomaterials contained in Ti-6Al-4V alloy powders (as representative multicomponent nanomaterials) and graphene, carbon-derived nanotubes (CNTs), or Ag-based metallic nanowires (as representative high aspect ratio nanomaterials) with emphasis on their physicochemical characterisation and their hazard, fate and risk assessment?*”.

### 2.2 Eligibility criteria

During this systematic review, studies which dealt with the representative MCNMs and HARNs as defined in the research question and which provided information on their physicochemical characteristics, potential property changes and transformations in complex environments, as well as one or more aspects of their hazard, exposure, fate, or risk assessment, were identified. Studies that did not report on either the physicochemical characterisation or their hazard, fate, or risk assessment (as applicable) were excluded.

To be considered for inclusion, the publications needed to be in English and published from January 2018 onwards. The reason for evaluating peer-reviewed publications from 2018 onwards is that this study aimed to capture the state-of-the-art regarding the rapidly evolving field of nanotechnology and novel and advanced nanomaterials like MCNMs and HARNs. This evolution is due to the emergence of new synthesis methods, advanced characterisation techniques, and better understanding of their applications in recent years, as well as a greater research focus on advanced materials as expressed in targeted EU and international public funding calls. This approach is further enhanced by the more detailed and in-depth insights into the hazard, fate, and risk assessment of nanomaterials, especially regarding their transformations in biological and environmental contexts. Literature post-2018 is likely to include research with more advanced methodologies for assessing the toxicological impacts and environmental transformations of complex NMs, and to be aligned with emerging and evolving SSbD principles and frameworks, and regulatory advances including the decision regarding nanoforms and groups of nanoforms [24].

### 2.3 Information sources and search terms

The source used for performing the literature search and subsequent review was Scopus [25]. The search was completed in December 2023. To maximise the retrieval of relevant literature, an optimised balance between a sensitive and specific search was required [26]. The difference between a sensitive and a specific search is the level of detail used for retrieving the results. A sensitive search is a generalised search, which allows the retrieval of a high volume of relevant information, while increasing the amount of irrelevant literature retrieved. This results in increased time required for filtering, screening, and reviewing the acquired literature [26]. A specific search is more focussed and leads to the retrieval of more relevant information, which leads to lower time requirements for literature filtering, screening, and reviewing [26]. The disadvantage of a specific search however is the increased probability of missing relevant literature due to the use of highly specific keywords compared to those used during the sensitive search process [26], [27]. For the current study, an example of a specific search targeted to the chemically doped ZnO multicomponent NMs is: multicomponent nanomaterials OR MCNMs AND ZnO AND rare earth elements AND hazard OR exposure OR release OR fate OR risk OR toxic OR adverse OR human OR environment. The respective sensitive search is: multicomponent nanomaterials OR MCNMs AND ZnO AND safety assessment. Here, a more specific approach was preferred since the intention was to focus on specifically selected representative MCNMs and HARNs and their hazard, exposure, and risk assessment. Thus, the searches performed were:

▪ Multicomponent nanomaterials OR MCNMs AND ZnO AND rare earth elements AND risk OR hazard OR exposure OR release OR fate OR toxic OR adverse OR human OR environment.
▪ Multicomponent nanomaterials OR MCNMs AND cerium zirconium mixtures OR Ce_x_Zr_y_O_2_ AND transition metals AND risk OR hazard OR exposure OR release OR fate OR toxic OR adverse OR human OR environment.
▪ Multicomponent nanomaterials OR MCNMs AND Titanium Carbide OR TiC AND Ti-6Al-4V OR TC4 OR Ti64 OR ASTM Grade 5 AND risk OR hazard OR exposure OR release OR fate OR toxic OR adverse OR human OR environment.
▪ High aspect ratio OR HARNs AND Graphene AND risk OR hazard OR exposure OR release OR fate OR toxic OR adverse OR human OR environment.
▪ High aspect ratio OR HARNs AND CNTs OR Carbon nanotubes AND risk OR hazard OR exposure OR release OR fate OR toxic OR adverse OR human OR environment.
▪ High aspect ratio OR HARNs AND Ag OR silver AND nanowires AND risk OR hazard OR exposure OR release OR fate OR toxic OR adverse OR human OR environment.

### 2.4 Study selection and analysis

**Fig. 2:**
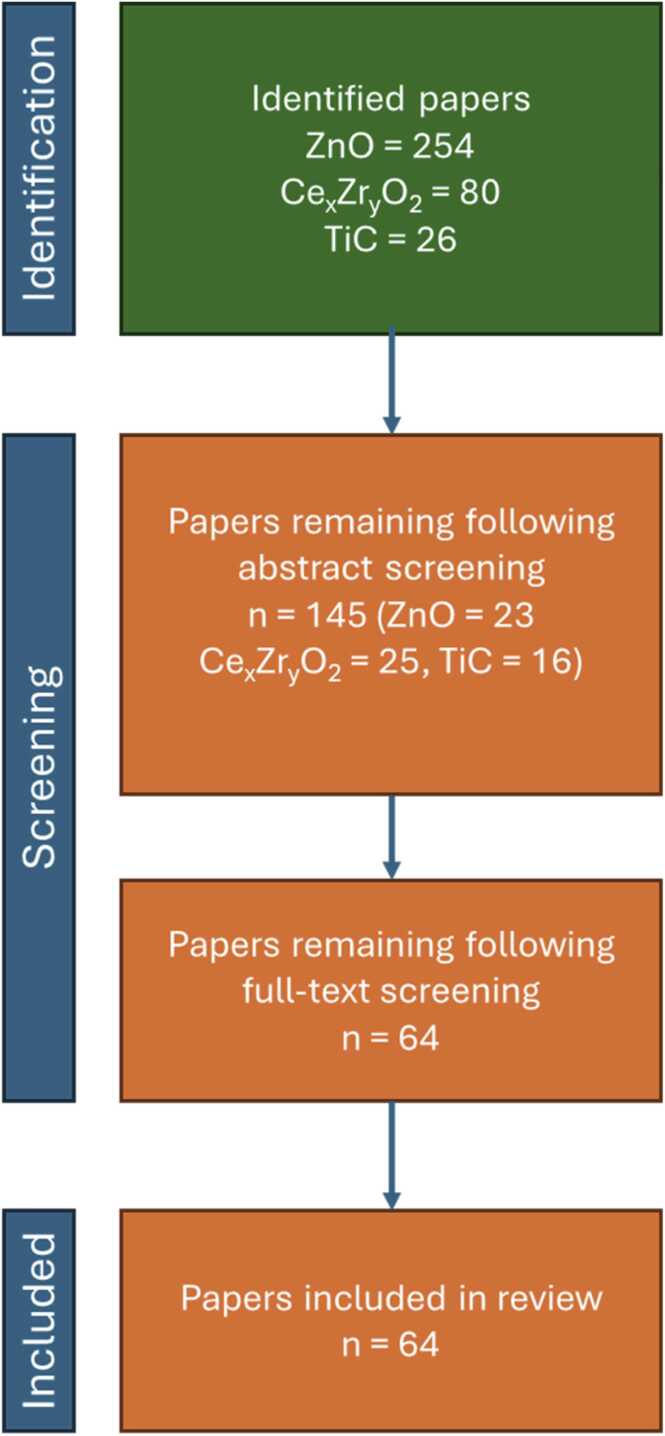
Summarised workflow and number of papers identified and evaluated at each stage of the systematic review process for the selected representative MCNMs (i.e., cerium-zirconium mixtures (Ce_x_Zr_y_O_2_), ZnO loaded with transition metals or rare earth elements, or Titanium Carbide (TiC) nanomaterials contained in Ti-6Al-4V alloy powders). Following screening and evaluation, a total of 64 MCNMs studies remained, and their full text was analysed.

Following the search in the Scopus database, the titles and abstracts of the retrieved papers were screened. Publications that did not meet the eligibility criteria or were not categorised as relevant, based on abstract screening, were removed. The full text of the remaining articles was carefully reviewed and analysed. The results were categorised based on the NM type and were grouped based on their focus to provide an assessment based on the 2 major segments, i.e., MCNMs and HARNs. A total of 358 papers was initially identified for MCNMs. From these, 143 were retained for analysis following abstract screening. From the papers, for which the full text was analysed, the NMs physicochemical characterisation was monitored to evaluate its completeness and if it was performed on the pristine, exposed, or both NM forms to identify potential changes in their physicochemical properties and transformations. The details of the respective hazard, fate or risk assessment were extracted and evaluated as well. The total number of papers fulfilling the criteria are 64 (ZnO doped with rare earth elements: 23, Ce_x_Zr_x_O_2_: 25, TiC-Ti6Al-4V alloys: 16). A summary of the workflow and the number of papers identified and evaluated at each stage for MCNMs is provided in Fig. 2. In the case of HARNs, a very large number of papers was identified (Graphene: n = 26,851, CNTs: n = 23,196, Ag nanowires: n = 8275). As it was not possible to screen and analyse all these articles, a different approach was followed. A total of 59 HARNs papers were selected (graphene: n = 20, CNTs: n = 24, AgNWs: n = 15), with the caveat that these covered all potential material uses, biological or environmental impact, and analysis. Focus was given, but was not limited to, to recent review papers to try and maximise the available body of knowledge.

### 2.5 Outcomes

This systematic review presents an overview on the state-of-the-art of the research performed for nanoscale cerium-zirconium mixtures (Ce_x_Zr_y_O_2_), ZnO NMs loaded with transition metals or rare earth elements, and Titanium Carbide (TiC) NMs contained in Ti-6Al-4V alloy powders (as representative MCNMs) and graphene, carbon-derived nanotubes (CNTs), and Ag-based metallic nanowires (as representative HARNs) regarding their physicochemical characterisation and their hazard, fate and risk assessment.

## 3 Results and discussion

### 3.1 Metal oxide MCNMs

Metal oxides are, currently, the largest NMs segment in the EU market providing around 75 % of the market value [2]. Metal oxide nano-containing products can be found in nearly all commercial segments employing NMs due to their versatile properties, especially their photoelectronic nature that enables them to be utilised in microelectronics, fuel cells, as coatings and catalysts, and in medicine, imaging, etc. [2]. Their usefulness is further enhanced via doping with rare earth elements, leading to optimised and finetuned photocatalytic and antibacterial properties [28], [29], [30]. From the commercially available metal oxides, ZnO doped with rare earth elements and zirconium doped cerium oxide NMs (Ce_x_Zr_y_O_2_ sometimes also indicated as Ce_1−x_Zr_x_O_2_) are being widely studied and used in different photocatalytic, antibacterial, and biomedical applications.

#### 3.1.1 ZnO MCNMs doped with one or more rare earth elements

ZnO nanomaterials are being widely used in nano-containing products due to their optical and antibacterial properties. ZnO is considered a strong photocatalyst as it is able to absorb most of the UV spectrum [31]. However, questions exist regarding their biological and environmental compatibility, as they may not be easily assimilated by the respective ecosystem following release, which can lead to toxic effects [32]. This is especially true in the case of catalytic applications using ZnO, especially near industrial photocatalytic applications or where ZnO is used in large-scale environmental remediation efforts. Furthermore, combining ZnO with other elements, e.g., by chemical doping, leads to changes in their properties like electron transfer, surface volume ratio, grain size, morphology, surface defect degree, and energy band structure, which also affects their behaviour [33], [34].

One approach to tailoring the properties of ZnO NMs is through doping with rare earth elements. Various synthesis methods have been used to produce doped ZnO NMs employing nearly all rare earth elements [28]. Synthesis and characterisation of ZnO hybrid nanostructures, e.g., with Yb, Eu, Nd, demonstrated increased visible emission following verification of the presence of ZnO nanocrystals doped with rare-earth ions using X-Ray Diffraction analysis (XRD) and Transmission Electron Microscopy (TEM) with Energy-dispersive X-ray spectroscopy [35], [36], [37]. The observed enhancement in visible emission was attributed to the increased radiative transition rates of dopants following incorporation into the crystal structure of ZnO NMs [35], as well as changes in the energy band gap (EBG) of the doped materials [36].

Doping with around 3 % of a rare earth element was demonstrated to be the most effective for the degradation of Malachite green dye using Sm-doped ZnO [38] and for Acid Red 17 textile dye using Dy-doped ZnO NMs [39], indicating that higher doping percentages form more electron–hole recombination sites that lead to decreased photocatalytic performance in spite of enhanced absorption [39]. Doping ZnO wurtzite (a semiconductor material) with rare earth elements, e.g., Gd, Er, led to crystalline defects and the introduction of surface oxygen defects had a direct effect on its luminescence, photocatalytic, and antibacterial behaviour [40]. ZnO doping with rare earth elements, i.e., Gd, Rb, La, Ce, leads to enhanced antibacterial activity, e.g., against *Escherichia coli*, *Pseudomonas aeruginosa*, and *Staphylococcus aureus* (*S. aureus*) due to the oxygen vacancies in the electron–hole pairs of the NMs leading to increased production of reactive oxygen species (ROS) [41], [42], [43]. Similarly, doping with Ce [44], Er [45], or Eu [46] has enabled the development of gas sensors for identification of ethanol, ammonia, and hydrogen, respectively, through the increase of oxygen vacancies and modification of the EBG that increased the gas sensing properties of ZnO NMs.

It has also been demonstrated that the presence of Ag NMs can enhance and optimise the sensitivity of luminescence-based Nd^3+^-doped ZnO based optical thermometers whereby the presence of Ag NMs increases the luminescence intensity due to local field effects and energy transfer from the metal NMs to Nd ions [36]. On the other hand, it is important to optimise the ratio of the doping [38], [39], as well as the doping relative to the presence of Ag NMs to avoid the reverse effects limiting the performance of such materials [36]. For Dy and Sm doped ZnO NMs, functionalisation with Ag NMs introduced a localised defect to modify the EBG of the doped ZnO NMs, and such defects determine the optical, structural, surface, and catalytic properties of the materials through variation of their morphology, elemental composition, and crystallinity [40].

While substantially more studies are being undertaken regarding rare earth element doped ZnO NMs and detailed characterisation of these materials takes place during their synthesis, detailed characterisation as part of exposure, fate, hazard and risk assessment studies, and where the doped NMs are contained within products or different environments is generally lacking. This is possibly because from a regulatory perspective doped NMs are considered to be equivalent to their pristine analogues under the REACH regulation in the EU (current as of July 2024) – while the introduction of nanoforms includes consideration of morphology and crystallinity, doping is not considered to change the material fundamentally, although we expect this position to change in the future as more complete understanding of MCNMs is collated, including that presented herein, Furthermore, since doping is applied specifically to modify the NMs properties, as noted in the examples above, the onus should be on producers to demonstrate that the doped materials behave similarly to the undoped parent and as such belongs to a set of nanoforms that includes the undoped parent).

**Fig. 3:**
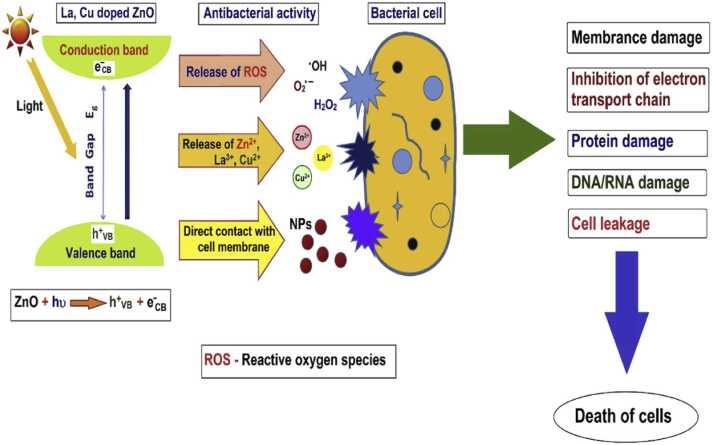
Schematic representation of the mechanism of action of La-Cu co-doped ZnO nanomaterials.

Reprinted with permission from [43], copyright ScienceDirect 2020.

Pristine ZnO NMs have been extensively tested regarding their adverse effects. While bulk ZnO demonstrates stability towards air, sunlight, and chemicals [47], the stability of ZnO NMs is less clear. It has been demonstrated that pristine ZnO NMs are unstable in biological media [47], [48], organisms, and plants [49], which leads to dissolution, the release of Zn^2+^, and ROS generation. ROS generation occurs due to the activation of the electron-hole pairs, which are able to disrupt the cell redox potential balance, or from crystallite defects [49]. This leads to toxic effects following several major pathways [49], e.g., disruption of the redox balance, activation of the p53 pathway which is a major tumour suppressor pathway that prevents the propagation of abnormal cells by regulating DNA repair, cell cycle progression, cell death, or senescence [50], or the nuclear factor NF-κB pathway leading to the expression of proinflammatory genes including cytokines, chemokines, and adhesion molecules. The drivers of ZnO NMs toxicity have been suggested to include properties such as size, shape, composition, surface charge and chemistry, surface acquired biomolecule corona composition, concentration, and route of exposure [47], [49]. For doped ZnO NMs, e.g., La-Cu co-doped Zn NMs [43], the main mechanism of action (Fig. 3) is through the decomposition of the NM leading to release of Zn^2+^ ions and ROS production..

**Table 1:** Toxicological effects of ZnO and doped ZnO NMs in in vitro and in vivo studies referenced in the current review.

**Material**	**Type**	**Target**	**Effect**	**Reference**
ZnO	*In vitro*	A549 cells (human lung carcinoma epithelial cells)	Cytotoxicity increases significantly with increasing ZnO NMs concentration to 1 mM	[47] (review) data from[51]
ZnO	*In vitro*	SH-SY5Y cells (human neuroblastoma cells)	Cytotoxicity increases significantly with increasing ZnO NMs concentration to 1 mM	[47] (review) data from[51]
ZnO	*In vitro*	Hs888Lu cells (human lung fibroblast cells)	50 % decrease in cell viability after 24 h incubation with 0.8 mM and 1 mM of ZnO NMs	[47] (review) data from[51]
ZnO	*In vitro*	U937 cells (pro-monocytic, human myeloid leukemia cell line)	40 %−50 % decrease in cell viability after 96 h incubation in 0.8 mM and 1 mM of ZnO NMs	[47] (review) data from[51]
ZnO	*In vitro*	RAW 264.7 cells (mouse macrophage)	IC50 of 12.16 – 22.08 μg/ml based on the assay for exposures of 24 −72 h	[47] (review) data from[52]
ZnO	*In vitro*	MH-S cells (mouse alveolar macrophage cells)	IC50 of 15.20 – 24.64 μg/ml based on exposures of 24 −72 h	[47] (review) data from[51]
ZnO	*In vitro*	Calu−3 cells (human lung epithelial cells from male adenocarcinoma patient)	No observed effect	[47] (review) data from[51]
ZnO	*In vitro*	16HBE cells (human bronchial epithelial cells)	IC50 of 17.79 – 56.46 μg/ml based on exposures of 24 h	[47] (review) data from[51]
ZnO	*In vitro*	TM3 cells (derived from Leydig cells from normal mouse testes)	IC50 of 6.16 – 10.06 μg/ml based on exposures of 24 h	[47] (review) data from[51]
ZnO	*In vitro*	TM4 cells (Sertoli cells isolated from testis of male mouse)	IC50 of 7.16 – 11.88 μg/ml based on exposures of 24 h	[47] (review) data from[51]
ZnO	*In vitro*	NIH/3T3 cells (mouse embryonic stem cells)	IC50 of 0.40 – 1.09 μg/ml based on exposures of 10 d	[47] (review) data from[51]
ZnO	*In vitro*	mES cells (mouse embryonic stem cells)	IC50 of 11.08 – 15.50 μg/ml based on exposures of 10 d	[47] (review) data from[51]
ZnO	*In vitro*	RAW 264.7 cells (mouse macrophage)	No significant toxicity for concentrations up to 100 μg/ml	[47] (review) data from[53]
ZnO	*In vitro*	Vero cells (derived from kidney of an African green monkey_	Cell viability decreased with increasing NM concentration	[47] (review) data from[54]
ZnO	*In vitro*	PK 15 cells (epithelial cells isolated from kidney of adult pig)	No significant effects up to 48 h for concentrations of up to 50 μg / 100 ml	[47] (review) data from[53]
ZnO	*In vitro*	MDBK cells (Madin-Darby canine kidney cells)	No significant effects up to 48 h for concentrations of up to 50 μg / 100 ml	[47] (review) data from[53]
Cu-doped ZnO	*In vitro*	E. coli	*E. coli* was reduced below detection limits	[48] (review) data from[55]
Fe-doped ZnO	*In vitro*	MSCs cells (murine mesenchymal stem cells)	2 wt% Fe doping was provided the best selective cytotoxicity between tumoral and normal cells.	[48] (review) data from[56]
Fe-doped ZnO	*In vitro*	BEAS−2B cells (non-tumorigenic human lung epithelial cells)	2 wt% Fe doping provided the best selective cytotoxicity between tumoral and normal cells.	[48] (review) data from[56]
Fe-doped ZnO	*In vitro*	KLN−205 cells (isolated from the lung of a mouse with squamous cell carcinoma)	2 wt% Fe doping provided the best selective cytotoxicity between tumoral and normal cells.	[48] (review) data from[56]
Fe-doped ZnO	*In vitro*	HeLa cells (epithelial cell derived from a cervical adenocarcinoma)	2 wt% Fe doping provided the best selective cytotoxicity between tumoral and normal cells.	[48] (review) data from[56]
Fe-doped ZnO	*In vivo*	Zebrafish embryos	Increased doping concentrations (up to 10 wt%) led to decreased adverse effects regarding the hatching rate	[48] (review) data from[57]
Fe-doped ZnO	*In vivo*	C57Bl/6NCrl mice	Increased doping concentrations (up to 10 wt%) led to reduction in pulmonary inflammation	[48] (review) data from[57]
ZnO	*In vitro*	HeLa cells	Internalisation and ROS generation	[58]
La-doped ZnO	*In vitro*	A498 cells	IC50 of 35.86 μg/ml at 24 h compared to 41.74 μg/ml for the pristine ZnO	[59]
La-doped ZnO	*In vitro*	Vero cells	IC50 of 49.69 μg/ml at 24 h compared to 55.27 μg/ml for the pristine ZnO	[59]
Ce-doped ZnO	*In vitro*	A498 cells	IC50 of 40.05 μg/ml at 24 h compared to 41.74 μg/ml for the pristine ZnO	[59]
Ce-doped ZnO	*In vitro*	Vero cells	IC50 of 56.83 μg/ml at 24 h compared to 55.27 μg/ml for the pristine ZnO	[59]
Nd-doped ZnO	*In vitro*	A498 cells	IC50 of 63.08 μg/ml at 24 h compared to 41.74 μg/ml for the pristine ZnO	[59]
Nd-doped ZnO	*In vitro*	Vero cells	IC50 of 51.10 μg/ml at 24 h compared to 55.27 μg/ml for the pristine ZnO	[59]
Y-Ce-co-doped ZnO	*In vitro*	HCT−116 cells	IC50 of 5.0–25 µg/ml	[60]
Y-Ce-co-doped ZnO	*In vitro*	HEK−293 cells	Cell viability was greater than that of HCT−116 cells	[60]

To overcome the challenges related to ZnO NM toxicity, doping has been proposed as an effective approach for reducing the observed toxicity [48], [49]. For example, Fe-doping of up to 10 wt% led to decreased toxicity, evidenced as improved hatching rate of zebrafish embryos and pulmonary inflammation in C57Bl/6NCrl mice, due to a reduction in dissolution and thus a reduction in release of ZnO^2+^ ions and consequent reduced ROS production [48]. Despite this, limited studies exist regarding the potential toxicity and other adverse effects of doped ZnO NMs, with current literature focussing mainly on anticancer strategies. Karthikeyan et al. studied the toxicity of La, Ce, and Nd doped ZnO NMs in A498 (human kidney carcinoma cells) and Vero (monkey kidney cell) lines, as well as antibacterial studies using gram positive (*S. aureus* and *Streptococcus pneumoniae*) and gram negative (*Klebsiella pneumoniae*, *Shigella dysenteriae*, *Escherichia coli*, *Pseudomonas aeruginosa*, and *Proteus vulgaris*) bacterial strains. The authors observed increased cytotoxic and antibacterial effects in the doped materials compared to the pure ZnO NMs [59]. Karthikeyan et al. concluded that substantial improvements, in terms of cytotoxic effects and cell specificity, were required for such materials to be applicable for therapeutic applications, despite the antibacterial effect observed against clinical pathogens from all materials, although La-doped ZnO exhibited the highest antibacterial effects [59]. Hannachi et al. studied the effect of Y-Ce co-doped ZnO NMs on cancerous HCT-116 and non-cancerous HEK-293 cells, as well as *E. coli* and *S. aureus* bacteria [60]. In all cases, increased toxicity was observed with co-doping, although cell viability was higher for the non-cancerous cells compared to the cancer ones [60].

In summary, doping of ZnO NMs with rare earth elements is being extensively studied for a range of applications, with the focus of current studies being on their photocatalytic and optical properties and approaches to enhance ZnO magnetism and electromechanical properties, followed by studies regarding their biocompatibility and use in medicine [48]. Despite the extended research, physicochemical characterisation is mainly performed on the as produced materials and studies monitoring potential changes and transformations of the doped NMs when exposed to different environments are lacking. Furthermore, the mechanism of action of such materials is still debated and clarity regarding the drivers of their behaviour and potential toxicity is unresolved [48]. This is especially significant in the case of doped ZnO dissolution, where the doping can potentially affect the physicochemical properties, their degree and rate of dissolution and subsequent adverse effects and toxicity. This poses an issue, especially considering the current REACH regulation that consider doped nanomaterials as equivalent to their pristine (undoped) analogues. This means that no further testing is required on the effects of the doping on dissolution, which reduces the potential impact of doping as a Safe by Design strategy through reduction of Zn^2+^ dissolution, but also ignores the potential for other not yet understood material transformations that may lead to potential toxic effects. Currently, conclusive data regarding the dissolution behaviour, and potential for cation exchange, of ZnO with rare earth elements are lacking, preventing the establishment of robust conclusions on whether the properties, dissolution, and behaviour of the doped analogues are equivalent to that of the pristine ZnO. Thus, further studies of doped nanomaterials, and further consideration by the regulatory authorities based on new data, are needed, including assessment of their dissolution, fate and effects in complex environments, organisms and the environment. Such studies should focus on comparing the dissolution rates of the pristine and doped analogues, the toxic potential of the dopant if released in its ionic form, and assess the long-term impacts of doped ZnO, including bioaccumulation and the potential for persistent and chronic toxic effects due to slow-release mechanisms.

#### 3.1.2 Cerium-zirconium mixtures (Ce_x_Zr_y_O_2_) as exemplars for transition metal doping

Cerium is one of the most abundant elements on earth and its oxide nanoforms are being extensively used in various applications like sensors, oxygen-permeable membranes, fuel cells, supercapacitors, cancer therapy, photocatalytic pollutant degradation, anticorrosion pigments, and polishing agents [61], [62]. CeO_2_ is a highly reducible substance, which reduces from Ce^4+^ to Ce^3+^ (redox cycle) leading to the formation of surface oxygen vacancies. While this redox cycling leads to CeO_2_ being a good catalyst, especially in the case of carbon degradation (e.g., as a catalytic converter for fuels), it can lead to unwanted behaviour due to its energy band gap overlap with the redox potential of cells. The catalytic activity of ceria NMs can be enhanced through doping [61] with transition metals, e.g., Zr, Co, Cu, Fe, which have been shown to lead to a broadening of its absorption range from the ultraviolet to the visible region of the light spectrum and alteration of the NMs respective photocatalytic activity [63]. This is achieved through changes to the ceria’s crystal structure and enhancement of the concentration of the oxygen vacancies, which exhibit a significantly lower optical band in the chemically doped NMs than in the pristine CeO_2_ NMs, making them safer and less toxic via oxidative stress mechanisms [63].

Other approaches regarding the use of transition metals with ceria NMs include the co-doping or loading (surface modification) of Ce_x_Zr_y_O_2_ with other transition metals or in the form of other oxides, e.g., a TiO_2_ shell layer. Cop et al. demonstrated the deposition of TiO_2_ into the mesoporous structure of Ce_0.5_Zr_0.5_O_2_ using atomic layer deposition, which was verified experimentally using Time-of-Flight Secondary Ion Mass Spectrometry (ToF-SIMS), Fast Fourier Transform (FFT) of high resolution transmission electron microscopy (HRTEM), and TEM-EDX [64]. This can allow the tailoring of the physicochemical properties of such systems through controlling the thickness of the active oxide layer, as demonstrated in a similar study using CeO_2_ deposition onto ordered mesoporous ZrO_2_ films that led to improved catalytic behaviour in oxidation reactions [65]. The usefulness of Zr-doping into ceria NMs has been demonstrated computationally using density functional theory (DFT) [66]. In this case, 50 % doping with Zr ions in the inner cationic sites was most energetically favourable, with the location of the Zr cations in the crystal structure being responsible for the system’s stability [66]. Koleva et al. demonstrated that a Zr core – Ce shell model, i.e., Zr cations substitute inner Ce ions, is the most energetically stable arrangement leading to higher oxygen mobility, which can enhance their catalytic performance [66]. The activity of these materials can be enhanced using co-doping or loading with transition metals leading to increased storage capacity [67].

A key catalytic application of such Ce_x_Zr_y_O_2_ materials is the synthesis of synthetic natural gas as a basis for production of carbon neutral fuels. This can be achieved through the methanation of CO_2_ and CO with hydrogen using Ce_x_Zr_y_O_2_-supported Ni catalysts [68], [69], [70], [71]. In this case, most of the Ni exists on the surface of Ce_x_Zr_y_O_2_ as bulk NiO and Ni nanoparticles. At the same time, part of the Ni may be incorporated into the Ce_x_Zr_y_O_2_ crystal lattice [71]. Development of Ce_x_Zr_y_O_2_ co-doped with praseodymium (Pr) impregnated with Ni led to methanation yields of up to 87 % at 300 °C [68]. This enhanced catalytic behaviour was attributed to different properties like “*high support metal interaction and reducibility, high surface area, and mesoporosity, linked with good Ni dispersion and surface area*” [68]. Evaluation of the effect of varying Ce/Zr molar ratios suggested that a ¼ ratio demonstrated the best basicity, i.e., the ability of a catalyst to activate CO_2_ that acts as an acid, which is important for reforming methane to generate renewable energy, and produces the most oxygen vacancies leading to higher methanation performance [72]. While environmental contamination from Ce_x_Zr_y_O_2_ NMs is relatively low, it could occur due to leaching during the use or disposal of catalytic converters and industrial processes [73].

Studies regarding the redox properties of these materials have demonstrated that the presence of Ni loading along with doping with Fe and Co lead to higher and more homogeneous reduction behaviour compared to the Ni-Ce_x_Zr_y_O_2_ materials [74]. The maximum methanation was observed for temperatures between 250 °C to 300 °C, with the greatest conversion achieved for the Ni-Co-Ce_x_Zr_y_O_2_ materials, while the Ni-Fe-Ce_x_Zr_y_O_2_ materials demonstrated higher conversion at temperatures below 250 °C [74]. This is due to the superior redox properties of Co, while Ce and Zr are excellent carriers for the methanation reaction, meaning they offer more active sites and thus higher CO_2_ conversion [74].

Loading of ceria-zirconia NMs with metal oxides has led to materials in which the transition metal oxides used, i.e., Mn, Cr, Fe, Cu, resulted in the uniform distribution of the Mn, Cr, and Fe oxide particles on the surface of the ceria-zirconia ones. On the other hand, CuO particle size was larger than the other oxides and comparable to that of the ceria-zirconia solid solution leading to a different interaction force as it was agglomerated on the surface of the particles [67]. In the study of Zhai et al., iron oxide loading led to nearly doubling of the oxygen storage capacity compared to that of the pristine ceria-zirconia NMs [67]. Cu, Cr, and Mn loading also demonstrated increased oxygen storage capacity, albeit lower than that of Fe. In all cases, a positive synergistic effect between the transition metals and the ceria-zirconia NMs was observed leading to a redox couple generated between the transition metals and the Ce ions, which can affect the oxygen status and improve the redox performance [67].

Cerium-zirconium mixtures loaded with Ru deposited on the surface have been shown to possess substantial catalytic properties for the selective oxidation of benzyl alcohol to benzaldehyde [75]. Ru incorporation into Ce_0.8_Zr_0.2_O_2_ without reduction using a 50 % H_2_/N_2_ gas mixture did not have any effect on the crystallite size of the NM, and XPS analysis and Rietveld refinement of the XRD diffractograms suggested that the Ru was dispersed on the surface of the Ce_0.8_Zr_0.2_O_2_ NMs in its metallic form [75]. The Ru-containing Ce_0.8_Zr_0.2_O_2_ NMs were found to have higher catalytic activity for conversion of benzyl alcohol to benzaldehyde and for the 1-phenylethanol secondary alcohol with 61 % and 55 % conversion at 90 °C, respectively, compared to around 30 % for the non-Ru containing NMs [75]. Similar results for Pt-loaded Ce_0.8_Zr_0.2_O_2_ NMs were obtained, whereby 100 % CO conversion was achieved at temperatures as low as 130 °C [76].

Lu et al. studied the catalytic performance of CuO-Ce_0.8_Zr_0.2_O_2_ catalysts doped with Co, Ni, Zn, and Mo transition metals [77]. The results demonstrated that CO and CO_2_ conversions significantly increased from 20 % and 45 % for CuO-Ce_0.8_Zr_0.2_O_2_ and Co-doped CuO-Ce_0.8_Zr_0.2_O_2_ NMs to 50 % and 95 %, respectively, with increasing temperature (80 °C to 100 °C). On the other hand, decreased conversion was observed for Ni-, Zn-, and Mo-doped CuO-Ce_0.8_Zr_0.2_O_2_ NMs [77]. This behaviour was attributed to Co substituting Ce leading to the formation of more oxygen vacancies, which increases the catalytic potential of the material. On the other hand, Ni substitutes Cu, while Zn and Mo are not incorporated into the material’s crystal structure but are dispersed on the surface of CuO-Ce_0.8_Zr_0.2_O_2_. In their case, no new oxygen vacancies were observed [77]. These results were further supported by Parastaev et al. who observed increased catalytic activity of Co co-doped Ce_0.5_Zr_0.5_O_2_ NMs during CO_2_ hydrogenation [78]. In this case, the increased activity was attributed to the presence of increased clusters of cobalt atoms along with cobalt oxide leading to an optimum adsorption potential of these materials [78]. In a subsequent study, Lyu et al. used transition metal oxide cluster deposition, NiO, CuO, FeO, NiCuO, and NiFeO, to enrich ceria zirconia mixtures to enhance the catalytic activity of such materials for the selective oxidation of methanol to methane [79]. In this case, the ceria-zirconia mixtures containing NiO demonstrated higher activity due to stronger Lewis acidity, which was further enhanced when Cu was added as well [79].

**Fig. 4:**
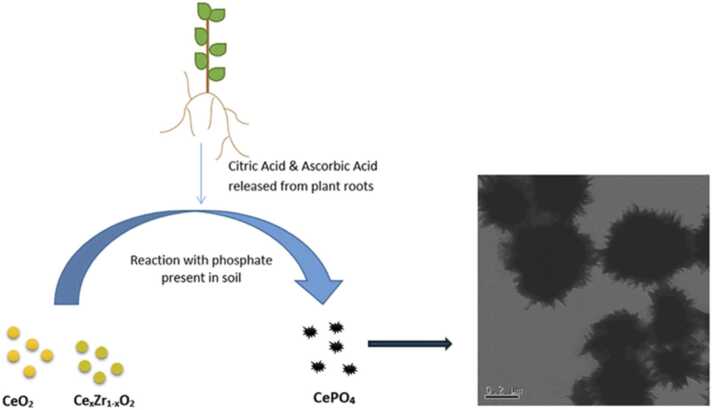
Cerium-based nanomaterials react with soil-present phosphates, which results in the nanomaterials’ physical transformation.

Adapted with permission from [80], copyright Elsevier 2019.

Despite the high interest and research in these materials, studies on their potential adverse effects and fate are lacking, while some studies exist regarding the toxicity of CeO_2_ NMs doped with different transition metals. This is despite the potential transformation of ceria-zirconia NMs following exposure to environmentally relevant media. Briffa et al. demonstrated that environmentally relevant phosphate concentrations induced transformations of zirconium-doped cerium dioxide NMs over a wide range of Ce:Zr ratios (Ce_1−x_Zr_x_O_2_, 0 ≤ x ≤ 1) [80]. Following exposure of these NMs to environmentally realistic phosphate solutions, e.g., monopotassium phosphate, citric acid, and ascorbic acid, Zr-doped ceria NMs transformed to larger and chemically different sea-urchin/needle-like particles (Fig. 4) of CePO_4_ / Ce_1−x_Zr_x_PO_4_ chemical composition. This transformation took place in a stepwise approach, starting with dissolution and reduction following exposure to citric and ascorbic acids. Following ion release, reprecipitation was observed with the Ce^3+^ ions reacting with the phosphate from the solution to form CePO_4_. The reprecipitation nucleated on the surface of the reduced NMs leading to the formation of the sea-urchin like structures and the significant increase in size observed [80]. Such transformations are possible if the materials are released into the environment and interact with, e.g., soil pore water during their lifecycle [80]. Briffa et al. concluded that while these transformations will affect the NMs’ bioavailability, the presence of phosphate phases will likely reduce their toxicity. On the other hand, the needle-like transformations may lead to increased physical damage, e.g., membrane puncture [80].

The cytotoxicity of Zr-doped ceria was studied by Naidi et al. who observed increased bactericidal activity against *S. aureus* for 1 % Zr-doped ceria NMs compared to the pristine (undoped) material and 5 % and 10 % Zr-doped ceria, which was attributed to Ce release and ROS generation [81]. Doping ceria NMs with other transition metals, e.g., Ag, Pd, has led to different outcomes [82], [83]. PEGylated Pd-doped ceria NMs were found to be less cytotoxic in a normal mouse NIH3T3 cell line than the undoped NMs, while they were more toxic for the A549 human lung carcinoma cell line due to ROS generation compared to the undoped NMs [82]. On the other hand, no significant cytotoxic activity was observed for Ce_0.99_Ag_0.01_O_2_, Ce_0.97_Ag_0.03_O_2_, and Ce_0.95_Ag_0.05_O_2_ NMs for concentrations up to 62.5 mM for the EPG 85–257 human gastric cancer cell line, above which a dose-dependent toxicity increase was observed [83] in agreement with other studies [84]. Similarly, a dose- and time-dependent cytotoxicity of Fe-doped ceria NMs has been observed by Rahdar et al. in MCF-7 breast cancer cell lines with an IC_50_ of 6.1 and 3.02 mg/ml following 48 and 72 h exposure, respectively [85]..

**Table 2:** Toxicological effects of doped CeO_2_ NMs from in vitro and in vivo studies referenced in the current review.

Material	Type	Target	Effect	Reference
Zr-doped CeO_2_	*In vitro*	*S. aureus*	10 % Zr-doped CeO_2_ demonstrated 52.7 % *S. aureus* biofilm inhibition compared to 73 % for the undoped	[81]
Zr-doped CeO_2_	*In vitro*	*L. monocytogenes*	10 % Zr-doped CeO_2_ demonstrated 56.9 % *S. aureus* biofilm inhibition compared to 59.8 % for the undoped	[81]
Pd-doped CeO_2_	*In vitro*	NIH3T3 cells	No significant cytotoxicity observed compared to substantial cytotoxicity for the pristine NMs	[82]
Pd-doped CeO_2_	*In vitro*	A549 cells	Increased cytotoxicity (50 % cell death at 150 μg/ml) compared to the pristine NMs at comparable concentration	[82]
Ag-doped CeO_2_	*In vitro*	EPG85 −257 cells	Higher cytotoxicity observed at 24 h for concentrations above 62.5 mM than that of the pristine NMs	[83]
Fe-doped CeO_2_	*In vitro*	MCF−7 cells	Reduction of cell proliferation and viability to about 21 % of the control after 72 h of treatment. No information on the pristine NMs was provided	[85]

#### 3.1.3 TiC nanomaterials contained in Ti-6Al-4V alloy powders

Ti-based composites, like Ti-6Al-4V, are being widely used in the automotive and aerospace industries [86], as well as in medicine, e.g., as bone and dental implants [87], [88], due to their robust physical and mechanical properties like fracture toughness, high strength, low elastic modulus, and low density. Ti-6Al-4V is among the most widely used alloys due to its excellent biocompatibility and mechanical properties. It presents an elongated grain structure, which mainly comprises of an α-Ti matrix with dimensions (length and width) in the range of a few micrometres [87]. The presence of submicron β-Ti phase has also been identified, which is uniformly distributed within the α-Ti matrix [87].

**Fig. 5:**
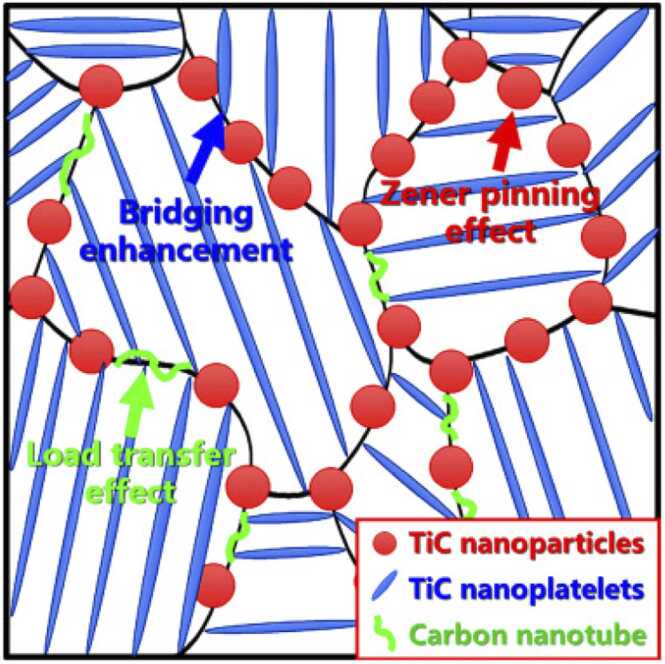
Schematic representation of the strengthening mechanism(s) of a CNT/TiC composite.

Reprinted with permission from [90], copyright Elsevier 2020.

Despite its excellent properties, Ti-6Al-4V can deteriorate over time due to the high wear rate originating from potentially load intensive applications. To overcome this wear, Ti-6Al-4V has been reinforced with NMs [86] to improve its properties and increase its lifecycle. Ceramic particles, like TiC, are considered to be among the best reinforcement agents due to their good wettability, high thermal stability, and similar thermal expansion coefficient to that of the steel alloy [86]. Embedding TiC NMs into Ti-6Al-4V alloys enhances their physicochemical and mechanical properties by inhibiting crack propagation in the α-Ti and reducing dislocation mobility to reinforce the β-Ti phases [86]. Among the methods used to reinforce Ti-6Al-4V alloys with TiC NMs, is that of the combined effect of nanoplatelets and nanoparticles (Fig. 5). Introducing TiC nanoplatelets leads to them being mainly distributed in the α-Ti phase of the alloy due to higher crystallographic similarity [86], [89]. Spherical TiC NMs, on the other hand, are mainly distributed in the β-Ti phase due to their higher diffusion [86]. Introducing TiC NMs in the Ti-6Al-4V alloy leads to respective structural changes. Ti-6Al-4V presents a “*near-equiaxed grain morphology*” with respective grain size refinement, which is attributed to the TiC nanoplatelet distribution inside the α-Ti phase or in the boundaries [86], [90]. The nanoplatelet orientation corresponds to that of the Ti-6Al-4V grain in which they are contained [90].

TiC-containing Ti-6Al-4V composites have been produced using various precursors like graphite powders, graphene oxide nanosheets, and graphene nanoplates [91]. Due to their higher defect content, graphene nanoplates were found to be easier to react with Ti-6Al-4V to form higher TiC contents that other carbon forms. TiC was again observed to connect to the Ti-6Al-4V matrix, as well as to interface with it, leading to better ductility and around 25 % higher yield strength than that of pure Ti-6Al-4V [91]. The preferential formation of carbides takes place at the defect sites due to the higher reactive nature of the carbon atoms in these sites, as is the case at the edges of the graphene nanoplates due to the oxidation-reduction fabrication process [91]. Furthermore, the development of Ti-6Al-4V alloys using processes that also lead to the formation of TiC can result in materials of increased hardness and decreased porosity due to the presence of both individual and agglomerated TiC particles, which have led to increased corrosion resistance and a decreased wear rate of up to 28 % [92], [93], [94].

The combination of TiC nanoplatelets and intragranular TiC NMs, i.e., where the TiC is deposited within the nanoplatelets grain boundaries, with carbon nanotubes (CNTs) leads to significantly increased hardness with increasing C-content [89], [90]. Li et al. observed a more than 500 MPa increase in yield strength at ∼10 % strain compared to the pristine Ti–6Al–4V alloy [90], following the addition of 0.25 wt% C during synthesis, while further increase of the C content led to a significant decrease in mechanical properties due to the synergistic effect of interfacial-distributed CNTs/TiC nanoparticles and intragranular-distributed TiC nanoplatelets [90]. A significant strengthening effect from embedding TiC NMs, in combination with CNTs, into the Ti–6Al–4V alloy was observed, which exhibited a good contraction with the Ti–6Al–4V matrix and reduced the amount of metallic debris following dry sliding wear tests compared to alloys without TiC [90]. TiC NMs and nanoplatelets were observed to be distributed along the grain boundaries and the intragranular microstructure, respectively, creating a honeycomb structure [90], [93], [94]. The TiC NMs and CNTs were assumed to strengthen the Ti–6Al–4V matrix by a load transfer effect, while the nanoplatelets enhanced the material strength via bridging enhancement and the Zener pinning effect, whereby TiC NMs prevent the motion of the nanoplatelets grain boundaries (Fig. 5) [90], [94].

Regarding the hazardous potential of such materials, aluminium, and vanadium release from Ti-6Al-4V is to be expected due to corrosion and wear. Vanadium has been linked with cytotoxic effects [95], which is a major concern considering its use in dental and orthopaedic implants. Thus, materials like TiC are used to enhance Ti-6Al-4V physicochemical properties and decrease potential corrosion and wear. It has been demonstrated that, e.g., in the case of dental and orthopaedic implants, that a Ti-6Al-4V/Ta_2_C alloy decreased the release of harmful ions, which is even further decreased with an extra MWCNT coating [88]. In fact, the presence of MWCNTs led to increased MG-63 cell growth and adhesion [88]. Similar results were acquired for graphene oxide/carbon fibres/polyetheretherketone coatings on Ti–6Al–4 V that reduced the in vitro cytotoxicity to murine fibroblast L929 cells, while demonstrating increased protection against *S. aureus* [96]. In general, studies have suggested that the use of carbon-based materials, e.g., graphene [97], and other non-toxic elements, e.g., Nb and Sn [98], to create Ti–6Al–4 V composites can lead to safer and more resistant materials. Considering the low cytotoxicity of TiC NMs [99] and their potential application as therapeutic agents [100], [101] demonstrates the potential of Ti–6Al–4 V/TiC composites..

**Table 3:** Toxicological effects of Ti-6Al-4V and TiC NMs in in vitro and in vivo studies referenced in the current review.

Material	Type	Target	Effect	Reference
Ti−6Al-4V	*In vitro*	MG−63 cells (fibroblast cells isolated from the bone of a human with osteosarcoma)	Poor surface cell spreading and proliferation	[88]
Ti−6Al-4V/Ta_2_C alloy	*In vitro*	MG−63 cells	Improved surface cell spreading and proliferation compared to Ti−6Al-4V	[88]
MWCNT-coated Ti−6Al-4V/Ta_2_C alloy	*In vitro*	MG−63 cells	Excellent surface cell spreading and proliferation compared to Ti−6Al-4V	[88]
GO/CF/PEEK-coated Ti−6Al-4V	*In vitro*	L929 cells (fibroblast-like cells derived from subcutaneous connective tissue of a male C3H/An mouse)	Significantly increased cell viability compared to uncoated Ti−6Al-4V	[96]
GO/CF/PEEK-coated Ti−6Al-4V	*In vitro*	S. aureus	Significant decrease of *S. aures* viability compared to control	[96]
Ti_3_C_2_T_x_	*In vitro*	3T3 cells	No significant impact on cell metabolism or induction of inflammatory pathways	[99]
Ti_3_C_2_T_x_	*In vitro*	Jurkat T cells (human T lymphocyte cells)	No significant impact on cell metabolism or induction of inflammatory pathways	[99]
Ti_3_C_2_T_x_	*In vitro*	THP−1 cells (human monocytic cell derived from acute monocytic leukemia patient)	No significant impact on cell metabolism or induction of inflammatory pathways	[99]

### 3.2 Carbon-based and Ag nanowires as exemplar HARNs

#### 3.2.1 Graphene materials

Graphene consists of a single layer of carbon atoms, which are arranged in 2D honeycomb (hexagonal) lattice [102]. It exhibits unique electrical, mechanical, and thermal properties making it a suitable candidate for use in various fields, e.g., electronics, energy storage and conversion, biomedical applications, and leading to extensive research. The widespread use of graphene has raised concerns regarding its hazardous potential, environmental impact, and potential toxicity [103], [104]. These concerns impact the scalability of graphene production and most importantly its regulatory acceptance and wider commercial use. Due to its popularity, there are numerous reviews regarding graphene, e.g., [105], [106], [107], [108], [109], [110], [111], [112], and here we present an overview of the state-of-the-art in graphene based NMs research and applications.

The integration of graphene into composite materials, as demonstrated with the TiC development in Ti–6Al–4V composites presented above, results in enhancements in strength, durability, and thermal and electrical conductivity. Such graphene-reinforced composites are making significant inroads in automotive, aerospace, and construction materials, offering the potential for lighter, more resilient structures [105], [106]. Graphene exhibits superior electrical properties and higher electron mobility than the currently used silicon analogues, which allows the development of electronic devices that faster and more energy efficient than their current state-of-the-art silicon counterparts [107], [108]. Thus, its use in field-effect transistors (FETs), ultrafast photodetectors, and flexible electronic components is being explored. Furthermore, graphene is at the forefront of next-generation energy storage and conversion devices, including batteries, supercapacitors, and solar cells [109], [110]. This is due to graphene’s high surface area and conductivity that offer improved charge storage capacity and rapid charge/discharge cycles, which play an important role for the development of high-performance lithium-ion batteries and supercapacitors [111], [112].

**Fig. 6:**
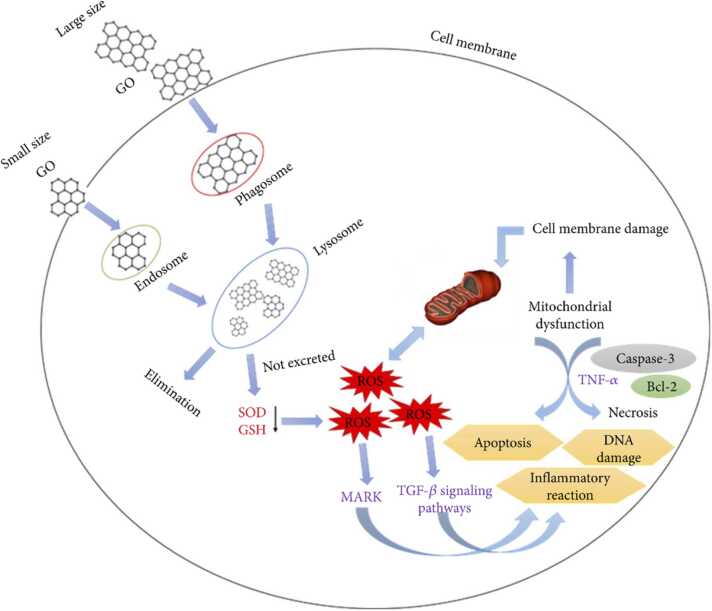
Schematic illustration of the toxicity mechanisms of graphene oxide.

Besides the potential safety concerns (that apply to all new materials and chemicals), graphene's biocompatibility makes it an exciting prospect for biomedical applications, including drug delivery systems, biosensors, and tissue engineering scaffolds. Its ability to interface with biological molecules can lead to breakthroughs in targeted therapies and diagnostics [113], [114]. Nevertheless, the special characteristics of graphene lead to complex interactions with organisms. As with other nanoscale, advanced, and complex materials, its behaviour is determined by its physicochemical properties including size, shape, surface charge, and functionalisation [115], [116], as well as the dose administered [103]. Studies have shown that graphene and its derivatives, e.g., graphene oxide, can induce cytotoxic effects (Fig. 6) or enhance the toxicity of other ions, e.g., Cd^2+^, through increased internalisation or by increased surface interactions [103]. As with other nanoscale, advanced, and complex materials, its behaviour is determined by its physicochemical properties including size, shape, surface charge, and functionalisation [115], [116]. Studies have shown that graphene and its derivatives, e.g., graphene oxide, can induce cytotoxic effects (Fig. 6). Graphene oxide can induce oxidative stress through, e.g., nicotinamide adenine dinucleotide phosphate oxidase (NADPH) dependent ROS formation and/or physical stress, while membrane damage can take place from the sharp edges of graphene sheets, and inflammatory responses through e.g., the disruption of mitochondrial electron transport chain [117]. The cytotoxic mechanism of action of graphene involves physical interactions with cellular membranes, disruption of membrane integrity, and induction of oxidative stress pathways [118], [119].

Reprinted under the Creative Commons Attribution License from [104].

The in vivo toxicity of graphene has been assessed in various animal models, e.g., rodents. In rodents, studies have demonstrated that graphene oxide can induce pulmonary fibrosis, an increased rate of mitochondrial respiration, ROS production, activation of inflammatory and apoptotic pathways [104]. Inhalation exposure of male mice (4 – 12 weeks old, from 2.5 – 5.0 mg/kg) and rats (6 – 10 weeks old, 0.54 - 10.1 mg/m^3^) to graphene materials has raised concerns over pulmonary toxicity, fibrosis, and granuloma formation [120]. From the studied materials graphene oxide seems to be the most toxic, followed by reduced graphene oxide, graphene nanoplatelets, and few layer graphene [120]. However, coating graphene oxide with an albumin corona led to reduced toxicity and improved biocompatibility, highlighting the role of surface modifications in mitigating adverse biological effects [115]..

**Table 4:** Toxicological effects of graphene-based materials in in vitro and in vivo studies referenced in the current review.

Material	Type	Target	Effect	Reference
Graphene oxide and functionalised graphene oxide	*In vitro*	Bacteria	Significant impact on bacterial metabolic activity, bacterial viability, and biological removal of nutrients	[103]
Graphene, graphine nano-powder grade C1	*In vitro*	Photosynthetic organisms	Dose-dependent toxicity observed	[103]
Graphene oxide and functionalised graphene oxide	*In vitro*	Photosynthetic organisms	Enhancement of toxicity through increased cell internalisation or surface reactions	[103]
Few-layer graphene, graphene oxide, functionalised graphene oxide	*In vivo*	Invertebrates	Bioaccumulation, reproduction, and growth rates effects	[7],[103],[114].[121]
Graphene oxide, graphene quantum dots	*In vivo*	Fish	Increased effects on growth inhibition, hatching delay, ROS, DNA damage, apoptosis with increasing dose	[103]
Graphehe oxide	*In vitro*	Human cell lines	Dose-dependent (5 −200 μg/ml) cytotoxicity, ROS, haemolytic activity	[104],[117]
Graphehe oxide	*In vitro*	Rodent cell lines	Dose-dependent (5 −200 μg/ml) cytotoxicity, ROS	[104]
Graphene oxide	*In vivo*	Rodents	Pulmonary fibrosis, increased rate of mitochondrial respiration, ROS, activation of inflammatory and apoptotic pathways	[104],[115],[120]
Graphne-based materials	*In vitro*	Biological membranes	Physical interactions, disruption of integrity, induction of oxidative stress pathways	[118],[119]

**Fig. 7:**
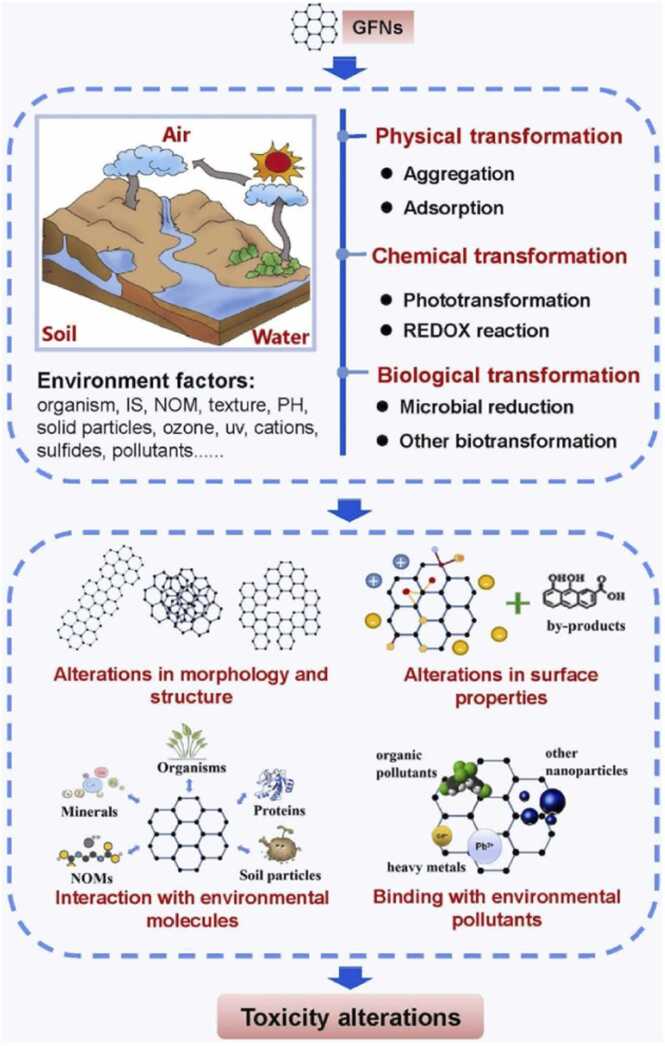
Potential environmental transformations of graphene based NMs and the mechanisms involved, which may result in alterations of their toxicity. GFN: graphene-family nanomaterials, IS: ionic strength, NOM: natural organic matter.

Reprinted with permission from [7], copyright Elsevier 2022.

The release of graphene into the environment, particularly aquatic ecosystems, poses potential risks to aquatic life. Studies have demonstrated that graphene and its derivatives can exert toxic effects on microorganisms, fish, and aquatic invertebrates, affecting cell viability, reproduction, and growth rates [7], [114], [121]. Graphene oxide has been found to induce cytotoxicity at relatively high concentrations, i.e., 200 μg/ml, although coating using PEG or BSA reduced the observed toxicity. On the other hand, hydrophilic graphene has been observed to induce ROS-related apoptosis in macrophages at concentrations around 50 μg/ml [121]. Similarly, administering graphite flakes or few layer graphene in mice at doses around 40 – 50 μg/animal led to lung inflammation. Inhalation of graphene nanoplatelets was not found to lead in any hazardous effects for doses up to 3.86 mg/m^3^ [121]. On the other hand, administration of graphene oxide and its derivatives, e.g., reduced graphene oxide, led to lung inhalation for doses as low as 18 μg/animal. In this case, during the end-of-life stages, graphene undergoes various transformations in the different environmental compartments (Fig. 7) and the derivatives may demonstrate different toxicity characteristics from the pristine particles [7]. These toxicity characteristics are attributed to changes in morphology, microstructure, and surface properties leading to diverse interactions with natural environmental molecules, as well as binding of environmental pollutants [7], [114]. Furthermore, understanding of the biodegradation and environmental fate of graphene is key for assessing its long-term environmental impact. Graphene may undergo limited biodegradation under environmental conditions, due to the presence of soil microflora and their enzymes, leading to it being oxidised to a graphene oxide-like material [122], raising concerns about its persistence and accumulation in ecosystems. The interaction of graphene with environmental matrices, such as soil and water, and its potential to bioaccumulate in food chains is an area that requires further investigation for the successful commercialisation of graphene.

Developing scalable synthesis methods that do not compromise the material's intrinsic properties is a key issue for the full exploitation of graphene. Techniques like chemical vapor deposition have shown promise for producing high-quality graphene, but issues with uniformity and cost remain [123]. Other issues, closely related to regulatory and safety considerations, include the need for standardisation of the techniques used to characterise and study graphene’s behaviour. Such techniques will need to be used in conjunction with developing comprehensive risk assessment frameworks to safeguard human health and the environment. Furthermore, current ISO and OECD standards for graphene and graphene-related materials focus largely on material characterisation and acute toxicity tests. but there is a gap regarding long-term exposure assessments. For example, while ISO/TR 10993–22 [124] provides some guidance on testing the biocompatibility of nanomaterials, it does not address the accumulation of graphene fragments in biological systems or their potential to cause chronic inflammation in organs like the lungs. While the biopersistence of graphene-based materials, their ability to interact with cellular membranes, and the potential for causing oxidative stress have been noted [125], standardised protocols for long-term environmental testing are lacking. Furthermore, the fragmentation of graphene during production, use, or disposal could result in the release of potentially toxic nanosheets into the environment, and their accumulation by organisms, or inhalation by humans, which current standards do not yet fully address.

#### 3.2.2 Carbon nanotubes

CNTs are cylindrical molecules consisting of rolled-up sheets of single-layer carbon atoms (graphene). They may consist of a single (single-wall) or multiple (multiwall) rolled-up sheet(s). Their crystal structure is analogous to that of graphite and diamond [126]. CNTs exhibit remarkable electrical, thermal, and mechanical properties, making them promising for numerous applications in electronics, optics, as biomedical agents, and for energy storage and conversion [126], [127]. As with other advanced materials, understanding their potential applications needs to happen parallel to studying their potential toxicity and environmental impact. Like with graphene, there are numerous reviews regarding CNTs toxicity [126], [127], [128], [129] and here we present an overview of state-of-the-art in CNT research and applications.

CNTs are being used as a key part of many composites to enhance their mechanical, thermal, and electrical properties [22]. CNTs enhance the tensile strength and durability of materials, without compromising weight and functionality [130], [131]. For example, incorporating CNTs into cement-based materials led to 30 % increased compressive strength and 50 % enhancement of the material’s flexural and tensile strengths [130]. Similar applications can be found in sports equipment [132], vehicle parts [131], and aerospace and defence engineering [133].

In electronics, research is focussing on developing transistors, substituting traditional silicon-based ones, that surpass the performance of, e.g., traditional complementary metal–oxide–semiconductor (CMOS) devices [134]. This has led to the development of 5 nm transistors used in integrated electronics [134] that can potentially enable faster and more efficient computers. While the integration of CNTs into more than 10,000 devices from different technologies has been achieved, there are several obstacles to overcome like standardising and automating material synthesis and processing control, device structure design and transport considerations [135]. Utilising metal nano-confinement, an emerging strategy to utilize the pores inside a support, whereby NMs that are themselves porous are embedded and thus confined by the support pores, CNTs have the potential to enhance the performance of batteries and supercapacitors, offering higher energy densities and faster charging times. In solar cells, CNT’s ability to form flexible, lightweight, and efficient photovoltaic elements is paving the way for next-generation solar power [136], [137]. A key obstacle to integrating CNTs in electrical and energy applications is the initial assessment of their design, functionality, and efficiency considering factors like cost, durability, performance, and environmental impact [136].

**Fig. 8:**
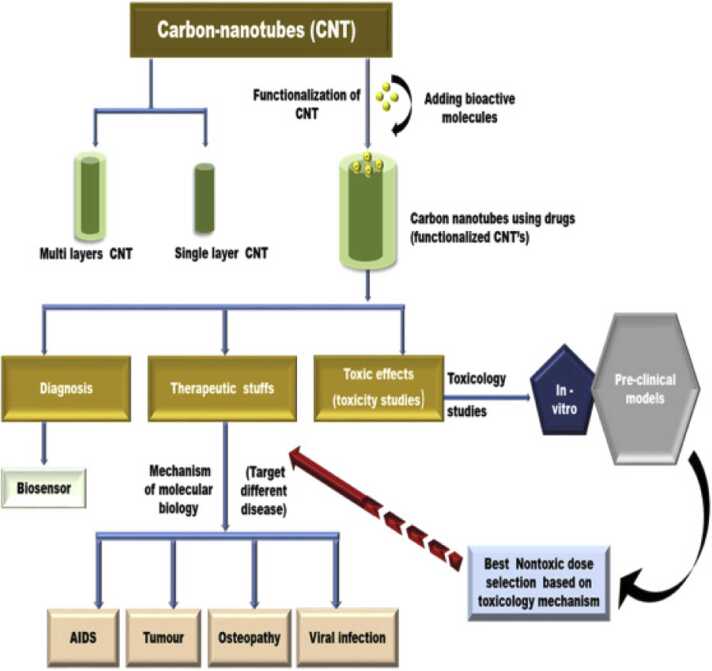
Schematic representation of the range of uses of CNTs in biomedical applications, and the need to assess their safety in parallel.

Reprinted with permission from [127]. Copyright Elsevier 2020.

Biomedically, CNTs are being explored for drug delivery [138], tissue engineering [139], genetic engineering [140], and as sensors for diagnostic purposes [141]. The application of CNTs in biomedicine (Fig. 8) is based on various properties of the CNTs. In genetic engineering, the structure, electrostatic properties, and vector capabilities affect how CNTs will interact with DNA molecules to facilitate gene delivery into cells, enhance DNA transfection efficiency, and protect DNA from destruction by cellular defence mechanisms [140]. CNT’s optical, electronic, and chemical properties can be enhanced through surface modification, e.g., functionalisation with Au NMs, to enable their use as high-brightness fluorescent probes and electron emitters for advanced imaging technologies [140], [141]. Surface functionalisation can also be employed for drug delivery, which takes advantage of specific physicochemical properties like their high surface area to increase the loading and needle-like structure to penetrate cell membranes and deliver drugs directly into target tissues, enhancing the effectiveness of treatments [140]. Furthermore, CNT’s biocompatibility, thermal, mechanical, and electrical properties promote their use as reinforcements in materials for implants and tissue regeneration, whereby they improve the mechanical properties of biomaterials and act as scaffolds for bone tissue [139], [140].

The interaction of CNTs with biological systems is complex and influenced by their physical and chemical characteristics. Certain forms of CNTs, specifically long and rigid ones, can induce adverse cellular responses, including oxidative stress and inflammatory reactions, leading to potential health risks as reported in various cell lines and animal models [129], although functionalisation could potentially mitigate these effects [126], [127].

A key concern regarding the hazardous potential of CNTs is their similarity to asbestos fibres, certain forms of which can lead to oxidative stress, chromosomal damage, and chronic inflammation, including through adsorption of toxicants and pollutants from their surroundings [142]. Like asbestos fibres, certain CNT types may enter non-phagocytic cells leading to mesothelial/epithelial cell injury [142]. The similarity of CNTs to asbestos fibres is attributed to their length and rigidity that can lead to common mechanisms of action potentially leading to pulmonary diseases [142]. Studies of SWCNT and MWCNT administration in mice have demonstrated that, like asbestos, the immune system is not able to clear rigid CNTs, longer than about 15 µm, which leads to “frustrated phagocytosis” [128]. As a result, reactions indicative of pulmonary inflation or granuloma due to blocked airways have been observed [128]. Studies in mice and rats have demonstrated peri-bronchial inflammation and necrosis, oxidative stress, and collagen deposition from the administration of SWCNTs. In the case of some MWCNTs, similar studies have shown effects like pulmonary fibrosis, granulomas, collagen depositions, and pneumonitis [129].

Recent developments in the EU’s regulatory framework have specifically focused on the classification of MWCNTs under the fibre pathogenicity paradigm (FPP) [143]. The Risk Assessment Committee (RAC) of the European Chemicals Agency (ECHA) adopted an opinion in 2022 (CLH-0–0000007108-75–01, adopted 18/03/2022), classifying MWCNTs with certain dimensions (length ≥ 5 µm, diameter ≥ 30 nm, and aspect ratio ≥ 3:1) as carcinogenic (category 1B) based on their biopersistence and specific dimensions. This classification is based on findings that these dimensions are critical in triggering mesothelioma and other lung pathologies [144]. MWCNTs with diameters < 15 nm are not included in this classification, as studies have indicated that smaller-diameter CNTs do not exhibit the same mesothelioma-causing potential [144]. This distinction is significant for ongoing risk assessments and regulatory decisions regarding smaller-diameter CNTs, as uncertainties exist regarding the hazardous potential of other CNTs, like those not meeting the FPP criteria [143]. For example, as stated in the comment by Wolff and Vogel [144], short and thin CNTs have been observed to cause bronchoalveolar hyperplasia and other lung conditions in animal studies, even though they do not align with the FPP’s carcinogenic criteria [144]. This suggests that further studies and regulatory evaluation are needed to identify potential health risks due to persistent inflammation from different types of CNTs.

As a result, large commercial and biomedical scale use of CNTs is lagging due to lack of complete understanding of how CNTs induce toxicity, which is not yet fully understood following several in vivo and in vitro studies [128]. Length, diameter, aspect ratio, rigidity, surface area, degree of aggregation, purity, concentration, functionalisation, structural modification, surface charge, impurities, and dose are among the CNT properties that have been linked with toxicity [128], [129]..

**Table 5:** Toxicological effects of CNTs in in vitro and in vivo studies referenced in the current review.

Material	Type	Target	Effect	Reference
Carboxyl, phenyl-SO34 or phenyl - (COOH)_2_ functionalised SWCNTs and MWCNTs	*In vitro*	Human dermal fibroblasts, THP−1 cells, alveolar macrophages from C57Bl/6 mice	Decreased cytotoxicity compared to pristine SWCNTs	[126], [127]
SWCNTs	*In vitro*	PC12 cells, HEK293 cells	Oxidative stress, increased cytotoxicity, apoptosis	[128]
SWCNTs, MWCNTs	*In vivo*	Rodents	Pulmonary inflammation, granuloma	[128]
MWCNTs	*In vitro*	HEK cells, Alveolar macrophages, A549 cells	Cell penetration, ROS, cytotoxicity,	[129]
SWCNTs and functionalised SWCNTs	*In vitro*	A549, HaCaT cells, HeLa cells, H1299 cells, HEK293T cells	ROS, cytotoxicity, cell proliferation inhibition, apoptosis	[129]
SWCNTs	*In vivo*	Rodents	Peri-bronchial inflammation and necrosis, oxidative stress, and collagen deposition	[129]
MWCNTs	*In vivo*	Rodents	Pulmonary fibrosis, inflammation, granulomas, collagen deposition, pneumonitis	[129]

Another issue that needs to be addressed is the environmental impact of CNTs, including their degradation and persistence. Currently, the study of CNTs in aquatic environments is challenging due to the lack of standardised methods for their repeatable and accurate preparation and characterisation in environmental media [145]. Furthermore, the existing approaches routinely applied to characterisation and quantification of metallic NMs, such as inductively coupled plasma mass spectrometry and reflectance-based imaging, are not applicable to CNTs due to their composition and physicochemical properties [145]. CNTs have the potential to accumulate in soil and aquatic environments due to their hydrophobic nature that leads to low dispersibility, although this can be overcome with the use of proper surface functionalisation, e.g., natural organic matter, hydroxide, and carboxyl groups, or through artificial means, e.g., sonication [145], [146]. The aggregation of CNTs with themselves (homoaggregation) or with other materials (heteroaggregation) will not only determine their persistence and transport through different environmental compartments, but their bioavailability and potential ecotoxicity [146]. This behaviour becomes more complex and harder to study at environmentally relevant concentrations making it hard to calculate and estimate the long-term environmental behaviour of CNTs, including their potential to bioaccumulate and enter the food chain.

Efforts to develop comprehensive risk assessment models for CNTs involve evaluating exposure scenarios across their lifecycle, from production to disposal. Occupational exposure during manufacturing processes has been a particular focus and a systematic review on the quality of occupational exposure assessments in field studies rated the majority of the included studies (n = 27) as moderate (n = 15) or low quality (n = 10) [147]. A key issue was the lack of methodological standardisation regarding the quantification of CNT agglomerates and/or CNT containing fibres, as well as an overly broad focus rather than specific cases that would allow a realistic evaluation of the CNT-related risks [147]. Thus, given the breadth and variety of materials classified as CNTs, development of guidelines for safe CNT production, handling, and use is hard to achieve. There have been several calls for differentiation between different forms of CNTs, and indeed the EU REACH regulations specification of nanoforms addresses this in part [148], [149], [150].

In summary, a significant challenge in CNT research is the lack of standardised methodologies for assessing their health and environmental impacts, making it difficult to compare results across studies. There is a need for longitudinal studies to understand the chronic effects of CNT exposure, as well as research into effective degradation or neutralisation methods to mitigate environmental accumulation. Additionally, as applications of CNTs expand, the anticipation, identification, and management of potential unintended consequences is required to ensure responsible and sustainable development. Currently, ISO and the OECD, through the Working Party on Manufactured nanomaterials, are working towards the development of protocols for the testing of complex and advanced materials, specific challenges remain. ISO 10808 [151] provides a method for measuring inhalation toxicity, but it does not fully capture the long-term biopersistence and frustrated phagocytosis mechanism that may contribute to pulmonary inflammation or carcinogenesis in the case of CNTs [144]. Additionally, OECD’s Testing Guidelines for nanomaterials do not comprehensively address the fibre-like behaviour of CNTs, some forms of which have been shown to mimic the hazardous characteristics of asbestos fibres in terms of shape, size, and durability [144].

#### 3.2.3 Ag nanowires

Like CNTs, silver nanowires (AgNWs) are distinguished by their high aspect ratio, with diameters in the nanometre scale and lengths up to several micrometres. This structure gives AgNWs distinct properties such as high electrical conductivity, thermal conductivity, and optical transparency. AgNWs are considered key for the development of optoelectronic applications due to their simple synthesis methods, their high chemical stability, and the ability to create networks with higher electrical conductivity compared to current silicon-based circuits [152]. These properties position AgNWs as a critical material in the development of flexible electronics, energy devices, and antimicrobial coatings [153], [154].

AgNWs are widely studied and used for the development of conductive inks and films for flexible electronics, including foldable displays, wearable sensors, and radio-frequency identification tags. Additionally, in the field of photonics, AgNWs are used to enhance the performance of light-emitting diodes and photodetectors through improved light absorption and scattering. This is due to their flexibility and conductivity, which enable the production of devices that can bend, stretch, and conform to dynamic surfaces without losing functionality [155], [156]. The performance of AgNWs is based on a combination of electrical and optical characteristics, stability, and mechanical properties [155]. One of their key characteristics is sheet resistance (also known as surface resistivity), which is influenced by the individual characteristics of the AgNWs, their interconnections, their aspect ratio, and overall distribution. However, challenges such as contact resistance (electrical resistance that arises at the contact point when components are connected) significantly impact the network's resistivity, highlighting the need for longer AgNWs to minimize contacts and resistance [155], [156]. The fabrication method affects the AgNWs' arrangement on substrates, altering sheet resistance. Techniques incorporating polymers, sintering, and welding can reduce resistance, but achieving uniformly conductive networks remains challenging [155]. Optical transparency is assessed by transmittance and the haze coefficient. Balancing electrical conductivity with light transmittance requires careful consideration of the AgNWs density, which inversely affects transparency [155].

Stability under various conditions, i.e., thermal, chemical, electrical, and environmental, is key for the longevity and functionality of AgNWs networks in devices. Employing protective layers, such as polymers or conductive ion gels, and composites enhances stability against degradation [155], [156]. Τhe mechanical properties of AgNWs, particularly surface roughness and flexibility, are vital for their application in optoelectronic devices. Reducing surface roughness and enhancing bending properties (flexibility) are essential for maintaining functionality under deformation, with strategies including the reduction of nano-junctions and the application of buffer and protective layers [155]. Similarly, high-temperature AgNW degradation is one of the key obstacles for fabricating AgNW-based flexible electronics, as AgNWs tend to decompose above 200 °C or when held at specific temperatures for extended time (> 20 min) periods [156]. In these cases, the AgNWs may breakdown into distinct Ag nanoparticles or see their sheet resistance increased [156].

AgNWs are also being used for energy harvesting and storage purposes. Their excellent thermal properties improve the efficiency of these devices and offer high conductivity and surface area, boosting their performance. AgNWs are key parts in the development of piezoelectric nanogenerators (PNGs) that aim to convert mechanical energy into electrical energy to power mobile and portable devices [157]. Flexible PNGs have been manufactured using composites of AgNWs with barium titanate embedded polyvinylidene difluoride composite films, where the AgNWs are being employed as a conducting supplement filler to improve the output performance [157]. AgNWs have also been also used in conjunction with triboelectric-piezoelectric hybrid nanogenerators (TPHNG) along with triboelectric-polydimethylsiloxane and piezoelectric- polyvinylidene fluoride in the form of a “fabricated sandwich” [158]. This structure demonstrates high transparency of 71 % along with good flexibility and is able to charge a commercial capacitor to 1.5 V within 100 s and to power an electronic watch [158]. TPHNGs are also able to detect the individual signals generated by body movements and can be used in wearable devices, while demonstrating increased stability under mechanical stress [158].

AgNWs have been studied for their antimicrobial properties against Gram-positive and Gram-negative bacteria [159], which are thought to be similar to those of Ag^+^ ions and silver nanoparticles, but with some differences in their mechanism of action, compared to ions and nanoparticles. AgNWs' antimicrobial activity is exploited in creating antibacterial coatings for medical devices, reducing the risk of infections. It is considered that the mechanism of action of Ag NMs is based on their surface area and the rate at which Ag^+^ ions are released. Thus, AgNWs having a high aspect ratio can be used to control antibacterial activity by affecting the rate of Ag^+^ release by engineering the size and length of the AgNWs to increase their surface area [159]. Further enhancement of the AgNWs activity can be achieved by loading these onto graphene oxide to prevent the AgNWs from aggregating, while enhancing their antibacterial activity. It has been suggested that the antibacterial effect arises from a combination of mechanisms, including increased ROS concentrations, leading to bacterial cell death, cell membrane disruption, and leakage of cellular contents [159]. .

**Table 6:** Toxicological effects of AgNWs in in vitro and in vivo studies referenced in the current review.

Material	Type	Target	Effect	Reference
AgNWs	*In vitro*	*E. coli*	Cell damage, protein disruption, ROS production, physical damage	[159]
AgNW treated polyethylene naphthalate surface	*In vitro*	Mouse embryonic fibroblasts	Increased cytotoxicity at 24 h	[159]
AgNW-loaded polydimethylsiloxane	*In vitro*	Human dermal fibroblasts	Dose-dependent cytotoxicity	[159]
AgNWs	*In vivo*	Rats	Dose-dependent pulmonary inflammation based on length and/or dissolution rates	[159]
PCL-loaded AgNWs	*In vitro*	C2C12 cells (mouse myoblast cellσ)	Increased cell proliferation	[160]
AgNWs	*In vivo*	*Artemia salina*	Increased ROS production and immobilisation (EC10: 0.036 mg/l, EC50: 0.43 mg/l) at 72 h compared to Ag NMs (EC10: 1.48 mg/l, EC50: 10.70 mg/l)	[161]
AgNWs	*In vivo*	*Chlorococcum infusionum*	Significant growth inhibition, decreased photosynthesis, reduced cell granularity	[162]
AgNWs	*In vivo*	*Caenorhabditis elegans*	Some observed toxicity compared to controls for concentrations above 5 mg/kg	[163]
Coated and uncoated AgNWs	*In vitro*	*Lettuce*	Diameter-dependent toxicity and accumulation	[164]

Ag-Au composites integrated into elastomeric block-copolymer matrices, result in composites with increased conductivity, while having optimised stretchability relative to the non-Ag-Au elastomeric materials. The composite structure also prevents oxidation and leaching of Ag^+^ ions, increasing its biocompatibility and allowing the development of implantable biosensors [165]. Similar results have been demonstrated through the development of AgNW/Polyvinyl butyral and hydrophilic polyurethane sponge (AgPHPUS) electrocardiogram electrodes that can be used for long-term monitoring of heart rate without leading to skin irritation or toxicity [166]. Finally, AgNWs can be used in tissue engineering as scaffolds to support cell growth and tissue regeneration. AgNWs loaded poly(ε-caprolactone) nanocomposites were found to lead to significantly more cell proliferation and cellular elongation compared to poly(ε-caprolactone) -only fibre scaffolds [160]. In this case, the AgNWs acted as conductive catalysts enhancing the electrostatic interaction between cells and the substrate [160].

AgNWs have also begun to be tested for their cytotoxic potential. While Ag nanoparticles have been extensively tested, this is not the case for AgNWs [159]. AgNWs have been found to be more toxic than Ag nanoparticles, in a dose-dependent manner in *A. salina* due to increased ROS production in cells exposed to the AgNWs [161]. Furthermore, studies on the bacterium *C. infusionum* demonstrated a toxic effect, which was correlated with AgNWs diameter [162]. By contrast, AgNWs were found to have a negligible effect, when comparison was made on mass basis, on growth and reproduction of *C. elegans* compared to Ag nanoparticles and nanoplatelets [163]. While Ag^+^ ion release has been reported to be the main driver of AgNWs toxicity, studies of PVP-coated and uncoated AgNWs on phytotoxicity and toxicokinetics of the dissolved and particulate Ag in terrestrial plants, showed particulate Ag to be the main reason for the observed toxicity [164]. Other studies have commented that AgNWs toxicity may be influenced by chemical interactions and transformations, e.g., sulfidation [159].

Silva et al. studied the effects of intratracheally instilled with 0.1 – 1.0 mg/kg of short (2 µm length) and long (20 µm length) AgNWs in male Sprague-Dawley rats [167]. While the animal weight was unaffected, the length, dose, and timepoint seem to significantly increase the Broncho-alveolar lavage (BAL) cell number and protein, while significantly producing neutrophilia and eosinophilia. No significant changes were observed in the macrophages number. Both AgNWs produced giant foreign bodies in the BAL macrophages, although only the long AgNWs produced frustrated phagocytosis [167]. A dose-response effect for the highest concentrations was also observed, which led to inflammation with the short AgNWs leading to worse responses over time. The responses included cellular exudate in alveoli, epithelial sloughing, Langhans cells in tissues, tissue granulomas, alveolar inflammation, bronchiolar inflammation, and particle-associated inflammation [167].

Based on the in vitro and in vivo results, it is evident that the mechanism of toxic action of AgNWs needs to be further investigated and clarified, making it a key experimental knowledge gap. Considering that AgNWs, like CNTs, may fall under the FPP paradigm [143] it would be desirable to evaluate whether fibre-like AgNWs, could pose similar health risks. Schinwald et al. demonstrated the FPP paradigm in Ag-nanowires (diameters > 100 nm) at a threshold of fibre length ≥ 14 µm for inflammatory response and restriction of macrophage locomotion for lengths ≥ 5 µm [168]. Silva et al., focussing on short vs. long AgNWs (diameter not reported), demonstrated a significant pulmonary effect for lengths as low as 2 µm in male Sprague-Dawley rats for intratracheally instilled AgNWs at doses of 0.5 and 1.0 mg/kg [167]. AgNWs, and indeed other fibre-like nanomaterials, may eventually trigger inflammation and fibrotic changes in lung tissue in a similar manner to CNTs [144], which means that biopersistence could be a factor in potential carcinogenicity. Thus, future regulatory evaluations may consider applying similar dimension-based assessments to AgNWs. The length, diameter, and biopersistence of AgNWs could play a significant role in determining their long-term health effects, particularly regarding their ability to trigger frustrated phagocytosis.

Decoding of their mechanism of action will help define the degradation processes of AgNWs in natural and biological environments that can assist with assessing their long-term impacts. Research is also needed to determine how AgNWs transform chemically and physically over time and their fate in different environmental compartments. To do so, there is a need for standardised protocols for testing AgNW toxicity and environmental impact, including the appropriate dose metric, facilitating the comparison of results across studies. Current approaches used for traditional NMs may not be directly applicable for HARNs and may require adaptations to the set-ups used to assess exposure to and impacts from wire-like materials. Regulatory frameworks must evolve to address the unique challenges posed by HARNs, ensuring safe production, use, and disposal of AgNW-based products. The currently available standards for NMs focus mainly on pristine Ag NMs and not on other forms like AgNWs. For example, ISO/TR 10993–22 [124] addresses the biocompatibility of nanomaterials, but there is insufficient guidance on the release of ions from HARNs like AgNWs and their potential hazardous effects. Similarly, current standards do not fully capture the long-term environmental behaviour of HARNs, including AgNWs. This is particularly true for ion leaching and the potential bioaccumulation of both silver ions and wire fragments in organisms. Thus, regulatory frameworks and guidelines must evolve to address the challenges posed by the increasing use of HARNs materials like AgNWs, ensuring safe production, use, and disposal of AgNW-based products.

## 4 Conclusion

This review has focussed on the state-of-the-art regarding the properties, applications, potential adverse effects, and challenges regarding specific MCNMs and HARNs. Although organisations like ISO and the OECD have made progress in creating guidelines for testing NMs, specific issues persist that require attention due to the complex nature of MCNMs and the fibre-like properties of HARNs. These challenges are directly linked to the structures, behaviour, and applications of the advanced materials discussed in this review.

MCNMs such as Ce_x_Zr_y_O_2_, ZnO doped with rare earth elements, and Ti-6Al-4V/TiC alloys require more detailed studies that consider their complex compositions, the way they transform in the environment, their fate, and potential effects when accumulated by organisms. For example, current REACH regulations treat doped materials as being equivalent to their undoped versions. However, as shown in this review, doping can significantly change the way ZnO dissolves and releases ions, which can impact its environmental and health risks. In particular, the presence of dopants can alter the dissolution behaviour and modulate the toxicity of ZnO, and indeed has been suggested as an SbD strategy to slow the dissolution of ZnO NMs, but current regulatory frameworks do not yet account for this possibility. Similarly, the fate of Ce_x_Zr_y_O_2_ NMs in the environment is not well understood. These materials are stable in their typical uses, such as in catalytic converters, but they can undergo chemical and morphological changes upon release to the environment or in biological matrices. This can influence their bioavailability and environmental impact.

For HARNs, including graphene-based materials, CNTs and AgNWs, the review has identified several gaps in understanding of the long-term health risks and environmental persistence. Although some progress has been made, especially with the classification of specific aspect ratio MWCNTs as carcinogenic under EU regulations, many other forms of CNTs remain unclassified. This is particularly true for shorter and tangled CNTs, which may still pose a risk for lung inflammation or chronic disease. The FPP, used for materials like asbestos, has been extended to cover certain types of CNTs, but it does not apply to all types of fibre-like NMs, meaning further research and more targeted regulations are needed to ensure that materials are classified appropriately. Similarly, AgNWs degrade over time, releasing Ag⁺ into the environment. These ions can be toxic, especially to aquatic life, but current testing standards do not fully evaluate the AgNWs persistence and long-term ion release. This presents a serious gap in the current safety assessments for these materials, particularly considering their widespread use in antimicrobial coatings and flexible electronics.

This review also highlighted gaps in the ISO and OECD standards that are relevant to the materials discussed. The existing standards tend to focus on short-term toxicity and acute exposure scenarios, and do not sufficiently address the long-term persistence of these materials or the potential for them to accumulate in living organisms. For instance, in the case of CNTs and AgNWs, the standards do not fully consider the risks associated with their biopersistence, i.e., their ability to remain in the environment or the human body for extended periods. Additionally, for materials like doped NMs including ZnO, the standards and current regulatory framing of nanoforms do not consider that doping may change the way these materials dissolve or release toxic ions, which could significantly affect their environmental behaviour and toxicity.

Research should focus on developing standardised methodologies and SSbD strategies to mitigate the potential toxicity and adverse environmental impacts of MCNMs and HARNs. This would enable the comparison of results across studies and facilitate the development of regulatory frameworks and *in silico* modelling approaches to reduce the need for animal tests in the future as part of the 3Rs directive’s goals of reducing, refining, and replacing animal testing. The implementation of SSbD strategies will also need the regulatory framework to evolve to allow differentiation between pristine and doped materials, for example, where the doping is applied to address a specific source of toxicity, such as dissolution.

The potential for modification of existing standardised guidelines for bulk substances and NMs should be considered, with the aim of developing workflows for design of inherently safe complex materials, including the use of biocompatible coatings, functionalisation to enhance biodegradability, and development of green synthesis methods. Additionally, establishing guidelines for the handling, use, recovery, and recycling or safe disposal of MCNMs and HARNs is critical to minimise occupational and environmental exposures.

## Funding

This research was funded by the European Union’s Horizon 2020 Research and Innovation Programme via the DIAGONAL (grant agreement nº 953152) and CompSafeNano (grant agreement nº 101008099) projects. The part regarding the nano catalysts was funded by the European Union Recovery and Resilience Facility of the NextGenerationEU instrument, through the Research and Innovation Foundation (Project: CODEVELOP-GT/0322/0093).

## CRediT authorship contribution statement

**Georgia Melagraki:** Writing – original draft. **Iseult Lynch:** Writing – original draft, Conceptualization. **Stavros Papatzelos:** Writing – original draft. **Dimitris Mintis:** Writing – original draft. **Anastasios G. Papadiamantis:** Writing – original draft. **Angelos Mavrogiorgis:** Writing – original draft. **Antreas Afantitis:** Writing – original draft.

## Declaration of Competing Interest

DGM, NC, AT, AGP, AA are affiliated with NovaMechanics, a cheminformatics and materials informatics company.
